# Leveraging AI techniques for predicting spatial distribution and determinants of carbon emission in China's Yangtze River Delta

**DOI:** 10.1038/s41598-024-65068-3

**Published:** 2024-07-04

**Authors:** Wen Zhang, Weijun Yuan, Wei Xuan, Yanfei Lu, Zhaoxu Huang

**Affiliations:** 1 School of Architecture and Art, Hefei University of Technology, Hefei, 230009 China; 2 School of Automation, Central South University, Changsha, 410083 China

**Keywords:** Climate change, Computational science, Computer science, Scientific data, Environmental impact, Climate-change adaptation, Climate-change impacts, Climate-change mitigation, Climate-change policy, Energy and society, Socioeconomic scenarios, Sustainability

## Abstract

This study focuses on the prediction and management of carbon emissions (CE) under the backdrop of global warming, with a particular emphasis on developing spatial planning strategies for urban clusters. In this context, we integrate artificial intelligence technologies to devise an optimized spatial analysis method based on the attributes of multi-source, urban-level spatio-temporal big data on CE. This method enhances both the accuracy and interpretability of CE data processing. Our objectives are to accurately analyze the current status of CE, predict the future spatial distribution of urban CE in the Yangtze River Delta (YRD), and identify key driving factors. We aim to provide pragmatic recommendations for sustainable urban carbon management planning. The findings indicate that: (1) the algorithm designed by us demonstrates excellent fitting capabilities in the analysis of CE data in the YRD, achieving a fitting accuracy of 0.93; (2) it is predicted that from 2025 to 2030, areas with higher CE in the YRD will be primarily concentrated in the 'Provincial Capital Belt' and the 'Heavy Industry Belt'; (3) the economic foundation has been identified as the most significant factor influencing CE in the YRD; (4) projections suggest that CE in the YRD are likely to peak by 2030.

## Introduction

As underscored in the Fourth Assessment Report by the Intergovernmental Panel on Climate Change, the principal catalyst for global warming is the significant emission of greenhouse gases, particularly CE^1^. Given the mounting challenges posed by climate change, the global urgency to develop strategies for mitigating CE has intensified^2^. As a significant global player, China has pledged to peak its CE by 2030 and achieve carbon neutrality by 2060. This commitment underscores the critical importance of accurate CE forecasting and management in the development of effective climate policies^3^. The rapid pace of urbanization has led to development that frequently extends beyond traditional administrative boundaries, complicating the management of regional CE. Traditional spatial governance models, based on these boundaries, are increasingly inadequate for the demands of sustainable urban development^4^. China’s '13th Five-Year Plan for Controlling Greenhouse Gas Emissions,' along with specific policies targeting urban agglomerations, underscores the interconnectedness of regional economic growth and carbon reduction efforts, particularly in densely populated urban areas such as the YRD^5^. The cities within the YRD span multiple provinces^6^ and are deeply interconnected in terms of economic and social development. This interconnectivity highlights both the exemplariness and the necessity of implementing unified and coordinated carbon reduction policies throughout the region^7^. Moreover, the complex makeup of these urban agglomerations complicates the acquisition and processing of necessary spatiotemporal big data for comprehensive analysis. The diversity of data sources, wide temporal ranges, and significant variability challenge the accuracy of urban data processing, thereby affecting the precision of policy formulation for CE reduction in urban clusters. Nevertheless, the onset of the digital era and advancements in AI technologies present new avenues for addressing these challenges^8^. Through the integrated analysis of multidimensional data that accurately reflects real-world conditions, particularly in fields such as Earth sciences and socio-economics^9^, AI technologies offer a more comprehensive quantitative analysis tool for managing CE in urban agglomerations^10,11^.

Considering the vital importance of CE forecasting and management in addressing global warming, this study zeroes in on the spatial planning of CE across 41 cities within the YRD using AI technology. This approach enables precise predictions of CE trends and identification of key influencers, thereby empowering policymakers to devise and execute more effective carbon reduction strategies. Such measures are crucial for laying a solid foundation for achieving long-term climate goals and ensuring sustainable development, in line with China’s dual carbon objectives and the significant influence of regional planning on the current dynamics within the YRD region and the practical application of existing technologies.

## Literature review

The selection of CE data, the identification of influencing factors, the methodologies used in spatial analysis of CE, and the potential applications of AI technology in this field have been extensively discussed by scholars worldwide at both national and city levels. Regarding CE and its influencers, the foundation for regional policy-making in CE planning is grounded in the analysis of historical CE data.

Currently, the primary datasets for CE are sourced from a diverse array of comprehensive global sources. These datasets enable the calculation of quantitative CE data, drawing from the Vulcan data within a broad emissions model, various national emission reports, satellite observations, and the China Emission Accounts and Datasets (CEADS)^12^. Scholars predominantly focus on the consumption of CE at regional and city scales. They examine the characteristics of CE consumption across various sectors including industry, transportation, construction, and household consumption^13,14^. It is also crucial to recognize that investigating the determinants of urban CE can provide valuable insights for addressing issues related to its reduction. Researchers have actively explored this area and identified that the primary factors influencing CE include economic growth, population growth, energy structure, and energy efficiency. These factors have been found to strongly correlate with fluctuations in CE^15,16^.

From a spatial analysis perspective, the disparities in resource endowment and stages of economic development among cities within an urban agglomeration theoretically result in significant spatiotemporal variations in CE intensity across different cities^4^. Existing case studies demonstrate a spatial correlation in consumption intensity among cities^15^. Therefore, it is crucial for CE planning to fully consider these disparities and correlations in spatial distribution^17^. Urban spatial data encapsulates information about the geographical location, inherent attributes, and distinct characteristics of cities within a spatial context^18^. Traditional models for spatial analysis include Nearest Neighbor, Inverse Distance Weighting, Kriging, Ordinary Least Square (OLS), Spline, Geographically Weighted Regression (GWR), GeoDetector, and various combinations thereof^8^. Extensive research has been conducted in this field. For instance, Lin et al. integrated spatial characteristics to advocate for the use of spatial lag models and spatial error models. They analyzed the CE space in the Guangdong-Hong Kong-Macao Greater Bay Area and its surrounding mainland cities, proposing key carbon reduction analysis strategies^19^. Chen et al.^20^ employed the GeoDetector model with optimal parameters to analyze the coupling coordination degree between PM2.5 pollution and CE emission reduction in the Yangtze River Economic Belt, revealing an upward trend in CE levels under conditions of multi-point agglomeration. Zhang et al. utilized the multivariate GWR method to dynamically simulate CE in the Pearl River Delta urban agglomeration, attempting to integrate complex CE scenarios with various governmental CE reduction policies, while accounting for spatial heterogeneity^21^. Overall, research predominantly focuses on the application of traditional spatial models in CE studies across various regions and scales.

However, traditional spatial analysis methods often encounter significant constraints when dealing with high-dimensional data, including computational complexity, overfitting, and the curse of dimensionality. These methods struggle to extract high-precision data patterns and conclusions from complex data sets. Such challenges highlight the need for the targeted development and application of more advanced spatial analysis techniques, specifically tailored to the data characteristics and spatial features of constrained real data. These innovative methods are essential to enhance the overall efficacy and precision of practical data utilization. The emergence of AI technology has significantly transformed the landscape of spatial analysis. AI technologies, particularly machine learning, have emerged as powerful alternatives to traditional prediction models. These technologies provide enhanced flexibility and can incorporate a broad range of covariates, including spatial information, without requiring additional computational effort^22^. Machine learning, which is not confined by linear relationships, is ideally suited for handling large volumes of high-dimensional or non-linear data, thus broadening the universality of the model^8^. It includes a variety of commonly used models such as Random Forest, Support Vector Machines, Decision Trees, Neural Networks, K-Nearest Neighbors, and CatBoost^23^. Current research has begun to compare various spatial prediction models across different applications. Some scholars have integrated spatial variables as feature variables into machine learning models, comparing the substitution effects of these models against traditional geographic models. Experiments have been conducted to predict variables such as temperature, soil metal concentration, air pollutant levels, and landslide conditions^24,25^. For example, Hengl et al. proposed a Random Forest spatial prediction method that uses the distance between all observations as covariates. These explanatory variables capture the spatial correlation of the response variables, enhancing the accuracy of predictions and yielding results comparable to the Kriging method^26^. These experiments primarily rely on the high fitting performance of machine learning to achieve more accurate data, albeit at some cost to interpretability. Some is on recognizing superior individual spatial correlations, which has significantly enhanced interpretability in spatial analysis. For instance, Zhang et al. utilized AI technology to refine techniques for identifying spatial relationships between individuals, leading to the development of the Back Propagation Neural Network. This approach was used to meticulously analyze the spatial relationships of urban blocks on an urban scale, providing precise block-level information essential for the scientific design standards of Changxing City^27^. A growing trend among scholars is exploring how AI technology can optimize urban CE spatial analysis. Based on the conducted analysis, this paper identifies several areas that merit further investigation:

1. Research Focus: Past spatial analyses of CE in China have predominantly concentrated on the southern regions, with relatively less exploration in the YRD area. The YRD region is the largest urban cluster in China in terms of its impact on CE, making it a highly necessary area for research.
2. Methodological perspective: Current research on urban spatial analysis primarily focuses on enriching the application scenarios of traditional geographic spatial models and enhancing their spatial precision. However, there is scant attention to how traditional and emerging AI models can complement each other to leverage their analytical strengths, thereby improving the precision and interpretability of data analysis.
3. Existing spatial analyses of CE largely focus on examining past scenarios. However, by forecasting future trends and identifying key influencing factors, we can provide substantial support for the development of effective carbon reduction, prevention, and management policies. This merits further investigation.

## Study area and data sources

### Study area

The YRD region, as China's largest urban agglomeration, encompasses approximately one-sixth of the national population and one-fifth of the total CE, contributing about a quarter of the country's GDP from just 4% of its land area. Positioned at the forefront of China's innovation and development, this area includes three provinces and one municipality, totaling 41 cities of varying sizes and functions, thereby creating a complex and integrated urban agglomeration. With the YRD's rapid development, environmental challenges, especially those related to sustainability and ecological health, have become increasingly pressing. In response, strategies emphasizing intelligence, low-carbon initiatives, sustainability, and coordination have been prioritized as core guiding principles for the region’s future planning and construction. Against this backdrop, precise CE forecasting and management within the YRD are pivotal in providing scientific bases and actionable guidelines for China to achieve its carbon peak and neutrality goals. These endeavors also offer valuable insights and strategies for other regions globally grappling with carbon reduction pressures in the context of climate warming, thereby holding significant leadership and exemplary potential in advancing regional low-carbon development strategies.

### Data sources

This study utilizes CE data, impact factor data, and geospatial data for cities within the YRD spanning from 2000 to 2019. The CE data are sourced from the CEADS, China's official repository for CE data, available at: https://www.ceads.net.cn/. CEADS was selected because it is a comprehensive, multi-scale CE accounting inventory, supported by authoritative bodies such as the National Natural Science Foundation of China and the UK Research Councils. CEADS is dedicated to developing a cross-verified multi-scale CE accounting system. The CE data it publicly releases has undergone rigorous academic validation and is widely used in leading international academic publications^28–31^. It provides comprehensive, officially recognized, and widely applicable CE data for Chinese cities, making it the primary source for our CE data research. At the same time, drawing on prior studies^20,21^, the factors influencing CE are classified into natural and socio-economic categories. This research selects 15 indicators across seven dimensions: natural factors, energy consumption, industrial structure, technological innovation, economic foundation, human capital, and governance capacity. Impact factor data for CE are obtained from the China Economic and Social Big Data Research Platform, primarily extracted from the < China Urban Statistical Yearbook > , available at: https://data.cnki.net/statisticalData/index. Among them, natural factors directly impact the mitigation of urban CE through mechanisms like photosynthesis. Energy consumption reflects the efficiency and cleanliness of energy usage in industrial processes, directly affecting CE. The industrial structure highlights a shift towards service-oriented and high-tech sectors, potentially reducing CE. Economic scale and growth rates are closely tied to both energy consumption and CE. Human capital affects the efficiency of energy use and the adoption of new technologies, influencing CE reduction. Governance capacity is crucial for formulating and implementing effective carbon reduction policies. Table 1 outlines the selection of these drivers and the interpretation of the variables. Map data was generated using the Gaode Map API, version 13.13.1.2025, available at: https://lbs.amap.com/. Due to the temporal scope of the data, missing entries were inevitable and have been addressed through interpolation in this study. Furthermore, because of significant variations in the values of different indicators, all data underwent maximum-minimum normalization before their inclusion in the calculations.

**Table 1:** Selection of driving variables for the CE in the YRD.

Categories factor	Code	Indicator layer
Natural factor	X1	Urban green space area
Energy consumption	X2	Volume of industrial wastewater discharge
	X3	Volume of industrial smoke and dust emission
Industry structure	X4	Relative contribution of the tertiary sector to the economy
	X5	Investment in scientific research and technological development
Economic foundation	X6	GDP
	X7	Total fixed asset investment
	X8	Aggregate investment in fixed assets
	X9	Total actual utilization of foreign capital
Human capital	X10	Mean remuneration of employed workforce
	X11	Education expenditure
	X12	Population
Governance capacity	X13	Comprehensive utilization rate of non-hazardous industrial solid waste
	X14	Centralized treatment rate of sewage treatment plants
	X15	Non-hazardous treatment of household waste rate

## Methods

To effectively address the challenges in urban CE analysis, it is crucial to delineate the spatiotemporal characteristics of existing urban-level CE data and to evaluate the strengths and weaknesses of current analysis methods. Such an understanding is essential for selecting appropriate solutions that align with the data characteristics, thereby facilitating the design of optimized approaches. In the process of identifying data characteristics, we employ global autocorrelation to explore spatial features, utilize the Pearson correlation coefficient to investigate interactions between datasets, and apply the OLS to assess the efficacy of existing spatial analysis models in recognizing regression relationships. Regarding method selection, we conduct a comparative analysis of traditional spatial analysis techniques versus emerging AI methods, focusing on data preparation, model development, and predictive capabilities(Table 2.). Based on this comparison, we decide to adopt an ensemble learning approach, which synergizes the strengths of various methodologies tailored to the identified data characteristics. Ultimately, this approach leverages ensemble learning to enhance traditional spatial analysis models through the integration of AI technology, addressing existing challenges such as low fit and limited interpretability. This strategy aims to more suitably address urban CE control issues for regional cities.

**Table 2:** Comparative analysis of different methods.

Method category	Example methods	Data preparation characteristics	Model development characteristics	Predictive accuracy characteristics
Traditional spatial analysis methods	GWR, Geodetector, Spatial Lag Models, etc	High demands on data characteristics; often requires extensive cleaning and processing. Struggles with high-dimensional data that has not been extensively cleaned, including nonlinear, small sample, and collinear datasets	Relatively simple to use	Poor predictive accuracy when data quality is low
AI algorithms	Catboost, Random Forest, Decision Trees, etc	Handles high-dimensional data effectively; however, the interpretability of results is often limited	Relatively simple to use	High predictive accuracy after proper parameter tuning
Ensemble algorithm designed in our study	CGWR	Effectively processes real-world data with small samples, nonlinearity, and collinearity while ensuring good interpretability	Utilizes stacking in ensemble learning, thoroughly validated. Users can directly apply the code from this paper for future predictions, regression relationship identification, and functional expansion	Significantly improved accuracy compared to both traditional spatial analysis methods and AI algorithms

### Methods for evaluating data characteristics

#### Assessment for data spatial characteristics

**Figure 1:**
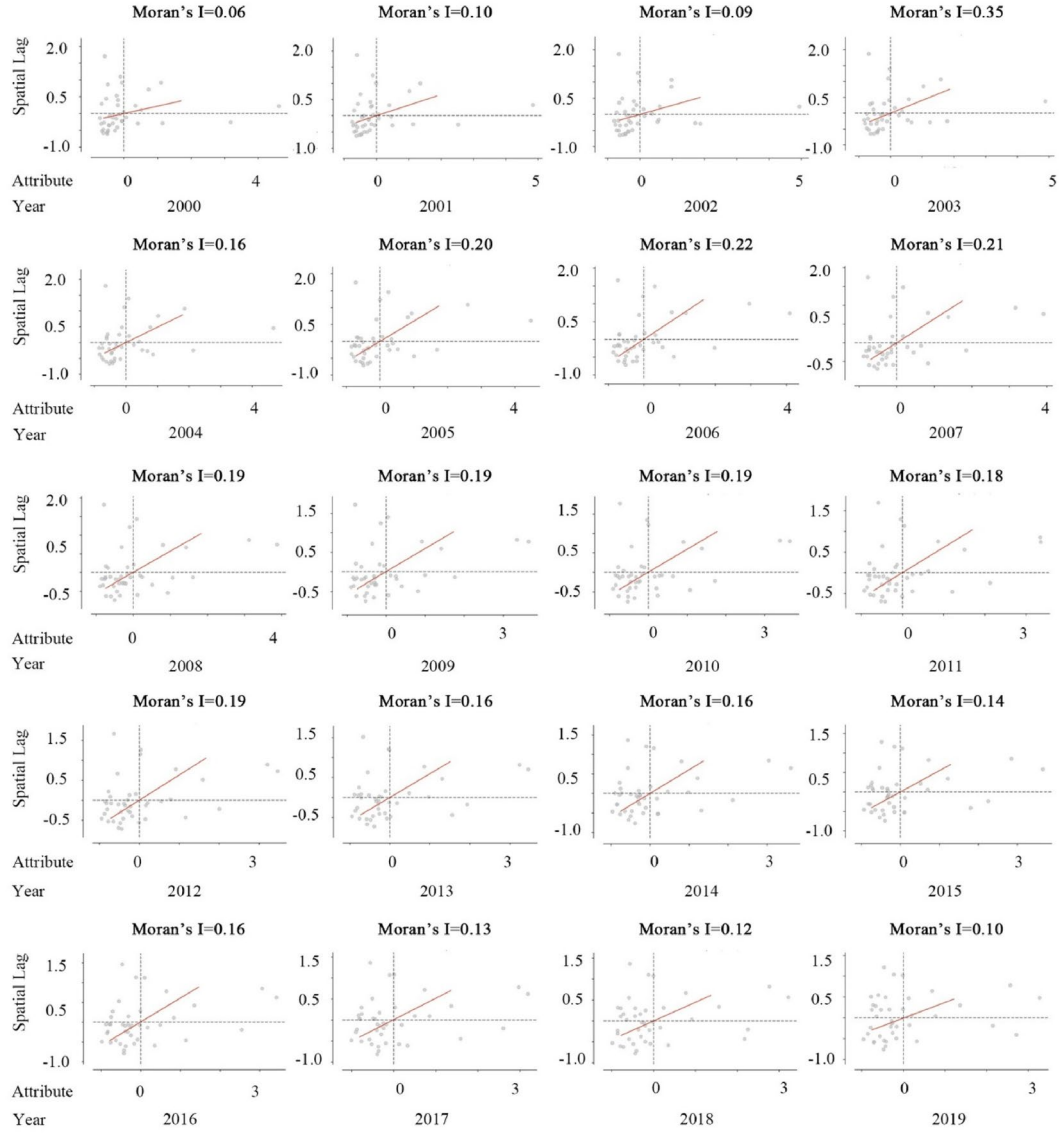
Scatterplot depicting the Global Moran’s I of CE in the YRD (2000–2019).

Spatial autocorrelation pertains to the spatial correlation of geographical phenomena, signifying the degree of similarity between neighboring regions. Moran’s I, a commonly used measure of spatial autocorrelation, spans a range from − 1 to 1. A value exceeding zero signifies spatial positive correlation or clustering, with larger values denoting more pronounced clustering. In contrast, a value less than zero suggests spatial negative correlation, with smaller values indicating more marked dispersion. A value of zero implies the absence of spatial autocorrelation^32^. The formula for Moran’s I is as follows:

1 $$\begin{array}{c}I=\frac{n{\sum }_{i=1}^{n}{\sum }_{j=1}^{n}{(Y}_{i}-\overline{\text{Y} })}{{\sum }_{i=1}^{n}{\sum }_{j=1}^{n}{w}_{ij}{\sum }_{i=1}^{n}{{(Y}_{i}-\overline{\text{Y} })}^{2}}\end{array}$$

where n is the total number of study subjects in the study area, ${w}_{ij}$ is the spatial weight value in the selected spatial weight matrix, ${Y}_{i}$ and ${Y}_{j}$ are the values of the study variables for the ith and jth study subjects in the study area, and $\overline{Y }$ is the average value of the study variables for all the study subjects in the study area. This study determines the spatiality of the CE in the YRD through global Moran’s I and finds that the value is greater than 0 from 2000 to 2019 (Fig. 1). This indicates that the results are all significant, and the CE of the YRD from 2000 to 2019 is not independent in space, but exhibits spatial autocorrelation. Therefore, this study needs to maintain and emphasize the accuracy and interpretability of the spatial information of CE data in the YRD.

### Assessment for data quality

In this subsection, we undertake a preliminary analysis of the assembled dataset to investigate the relationships among the influencing factors and between these factors and the dependent variable, namely, CE. This study denote the factor set as $\text{X}=\left\{{\text{x}}_{1},{\text{x}}_{2}, ...\left., {\text{x}}_{20}\right\}\right.$, encompassing all the factors described in this paper, where each ${\text{x}}_{i} (i=\text{1,2},\dots ,20)$ represents an individual feature.

#### Covariance assessment for data quality

**Figure 2:**
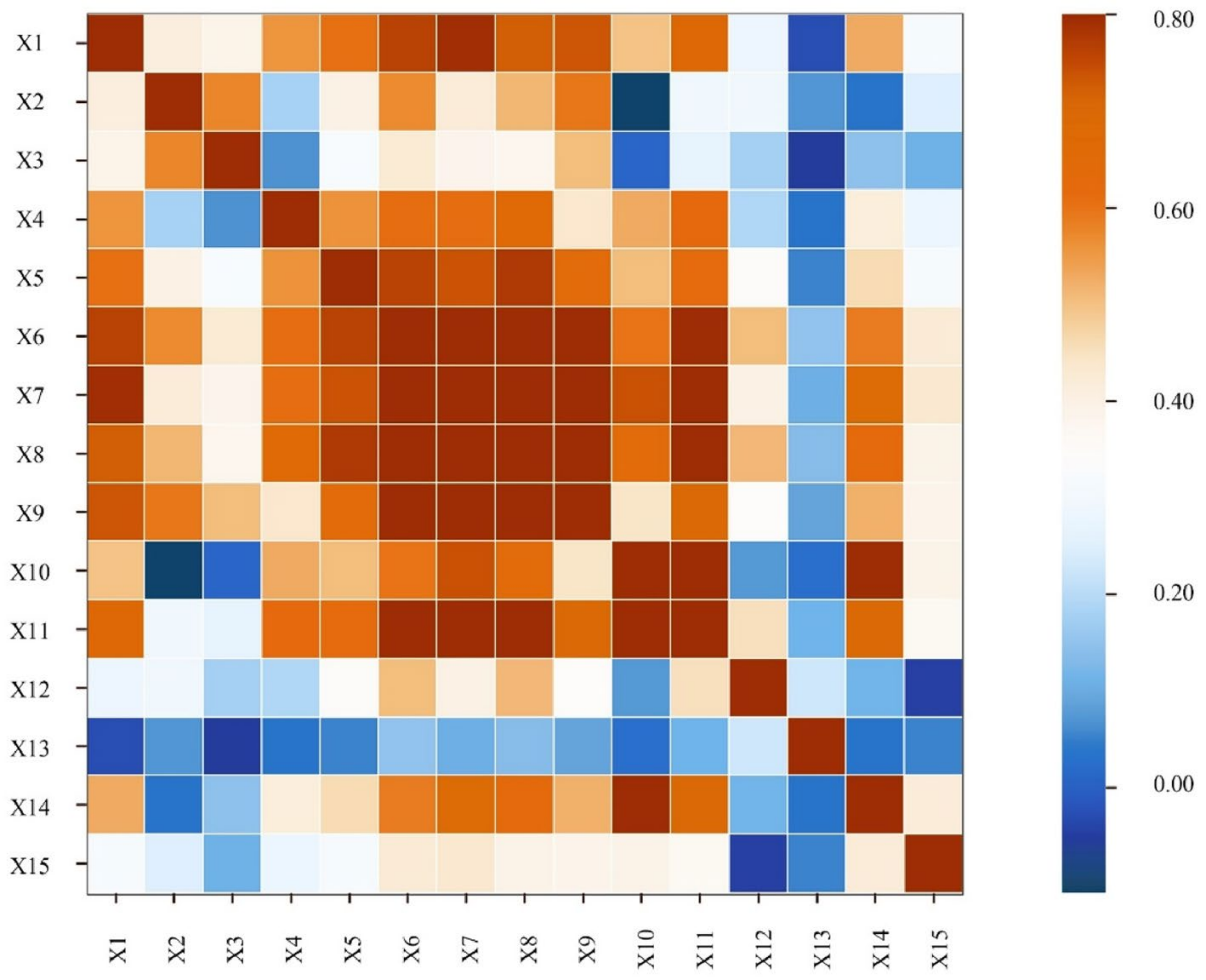
Collinearity identification among influencing factors based on Pearson correlation coefficient.

During computational analysis, independent variables may display covariance, characterized by intercorrelation among variables. While moderate covariance has minimal impact, severe covariance, if not adequately addressed, can destabilize analysis results and compromise their reliability. The covariance of the data can be assessed by examining the correlations between features. In natural sciences, the Pearson correlation coefficient is commonly used to determine the extent of correlation within data^33^. Pearson correlation coefficient ranges from − 1 to + 1, where − 1 signifies a perfect negative correlation between the influencing elements, + 1 denotes a perfect positive correlation, and 0 indicates no correlation. This study investigates the relationships between various influencing factors from 2000 to 2019 using the Pearson correlation coefficient. The results, depicted in Fig. 2, reveal that correlation coefficients between several independent variables exceed 0.60, indicating a strong correlation among them. Consequently, this paper identifies severe multicollinearity among the factors, which must be addressed during the computation process. This issue may be attributed to the small sample size, a common challenge in collecting yearbook data, and the tendency for phenomena to not evolve independently.

#### Nonlinear assessment for data quality

**Figure 3:**
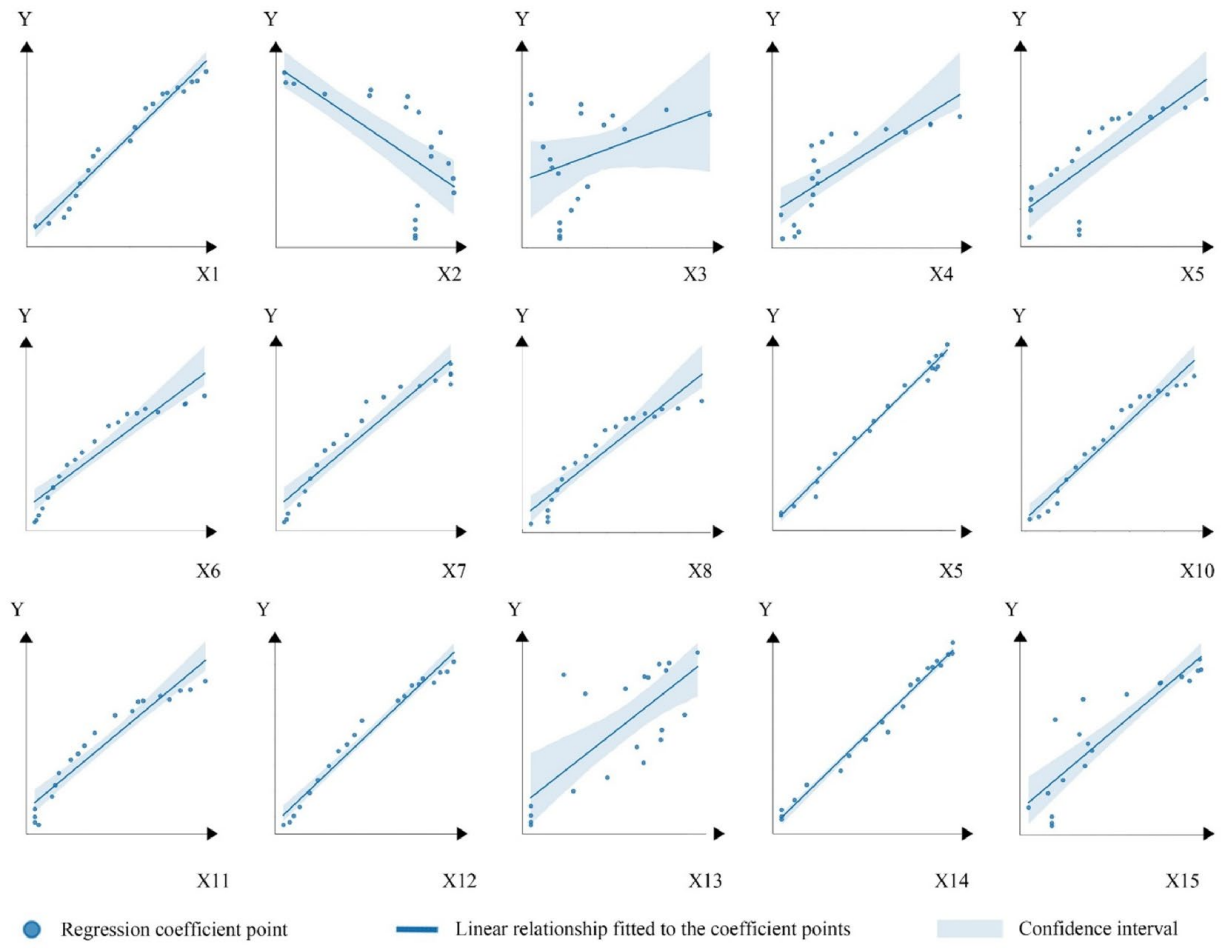
Identification of the linear relationship between influencing factors and CE based on the OLS method.

Traditional geographic models often utilize the OLS method, which identifies linear relationships between variables. To further investigate the linear fitting status of the data, this study employs the OLS method to conduct a preliminary experiment examining the relationship between various factors and CE, as illustrated in Fig. 3. The scatter points in Fig. 3 represent the observed regression coefficients between the mean city feature values (horizontal axis) and the mean city CE values (vertical axis) from 2000 to 2019, with the confidence interval depicted in blue. The line represents the linear regression relationship identified through the OLS method. Figure 3 reveals a non-linear relationship between multiple factors and the dependent variable, indicating that the fitting effect of the traditional linear model is suboptimal. This may be attributed to challenges such as difficulty in collecting yearbook data, a small sample size, and the fluctuating nature of the actual situation due to policy changes.

To specifically enhance data processing precision, identifying data characteristics is a crucial preliminary step. Figures 1, 2, and 3 initially display the spatial distribution, covariance, and non-linear relationship analyses of CE and their influencing factors in the YRD. These figures highlight evident spatial patterns and covariance among variables, as well as typical non-linear interactions in small-sample scenarios. Clarifying the spatial, covariance, and non-linear properties of the data is essential for understanding how these factors impact model fitting. These characteristics significantly influence the accuracy and reliability of geographical spatial analysis models. Subsequently, we selected algorithms tailored to address these specific data characteristics. Figures 5 and 6 also demonstrate that using targeted algorithms to address these issues significantly enhances the precision of the results.

### Methods for algorithm design

In terms of methodology design, we initially conduct a comparative analysis of traditional spatial analysis methods and emerging AI techniques, focusing on data preparation, model development, and predictive capabilities. Subsequently, based on the characteristics of our data and processing requirements, we devise our methodology by integrating the strengths and compensating for the weaknesses of these approaches. We then comprehensively compare the effectiveness of the various methods employed. The comparative analysis of these methods is summarized in the Table 2.

Ultimately, after systematically comparing various algorithms and their synergistic effects in a standardized manner using Python software, this study employs two specific algorithms, GWR and CatBoost, utilizing ensemble learning techniques for stacking. This research focuses on applying these sophisticated techniques to effectively address practical issues, rather than delving into the theoretical design of the algorithms themselves. Consequently, this paper briefly outlines the selection and calibration principles of the chosen algorithms for experimental purposes, placing particular emphasis on ensuring both data accuracy and spatial interpretability.

### GWR

GWR is a spatially autocorrelated weighted regression model that has become a widely used classic model for analyzing spatial relationships and influencing factors of geographic phenomena. This model enhances the traditional OLS regression by incorporating spatial location information into the spatial regression framework, thereby addressing the issue of relationship or structural changes between variables that occur due to geographic location changes. GWR effectively accounts for the spatial heterogeneity and spatial correlation of data, providing high interpretability and reliability when dealing with spatial features. However, despite its strengths, GWR remains fundamentally a linear regression model and may encounter issues of poor fit in non-linear scenarios. The formula for GWR is as follows:

2 $$\begin{array}{c}{Y}_{i}={\beta }_{0}\left({{u}_{i},v}_{i}\right)+{\beta }_{1}\left({{u}_{i},v}_{i}\right){X}_{i1}+ \cdots +{\beta }_{p}\left({{u}_{i},v}_{i}\right){X}_{ip}+{\varepsilon }_{i}, i=\text{1,2},\cdots ,n\end{array}$$

where ${Y}_{i}$ is the observation of the dependent variable at geographic location $({{u}_{i},v}_{i})$, ${X}_{i1},\cdots ,{X}_{ip}$ is the observation of the independent variable at geographic location $\left({{u}_{i},v}_{i}\right)$, ${\beta }_{j}(u,v)(j=\text{0,1},2, \cdots p)$ is an unknown function of the spatial geographic location (u ,v), and ${\varepsilon }_{i} (=\text{1,2}\cdots ,n)$ is an independently and identically distributed random perturbation term that satisfies the criteria of $E\left({\varepsilon }_{i}\right)=0$, $Var\left({\varepsilon }_{i}\right)={\sigma }^{2}$.

### CatBoost

Machine learning, a branch of AI technology, utilizes mathematical models to enable computers to learn specific tasks. The gradient boosting technique, a machine learning method, has become the primary approach for addressing learning challenges such as heterogeneous features, noisy data, and complex dependencies in recent years^34^. CatBoost, a robust gradient boosting algorithm, has demonstrated its efficacy in handling non-linear, covariate, and small sample data in both existing literature and simulation experiments. The reasons why it can handle these data features well are as follows: firstly, CatBoost exhibits the capability to automatically capture non-linear relationships when dealing with noisy data, eliminating the need for manual feature engineering or polynomial feature expansion. Its tree model structure makes it adept at handling non-linear relationships^34^. Secondly, in terms of covariance, CatBoost mitigates this data issue through powerful regularization techniques^35^. Thirdly, CatBoost introduces two critical algorithmic advancements compared to other gradient-boosting-based algorithms. The first is the implementation of ordered boosting, a permutation-driven alternative to the conventional algorithm. The second innovation pertains to the handling of categorical features, where CatBoost employs a novel algorithm for more effective processing^34^. With these improvements, CatBoost has demonstrated strong performance on limited samples of certain datasets^36–39^. The steps of CatBoost used in this study are as follows.

For a given dataset $\mathcal{D}={\left\{\left({\text{x}}_{k}, {\mathcal{y}}_{k}\right)\right\}}_{k=1, 2, \dots , n}$, where ${\text{x}}_{k}=\left({x}_{k}^{1}, \dots , {x}_{k}^{m}\right)$ is a vector of $m$ features and ${\mathcal{y}}_{k}\in {\mathbb{R}}$ is a $target$, which could be either a label or a numerical response. The examples $\left({\text{x}}_{k}, {\mathcal{y}}_{k}\right)$ are drawn independently and randomly from an unknown distribution $P(\cdot , \cdot )$. The objective of a learning task is to train a function $F:{\mathbb{R}}_{m}\to {\mathbb{R}}$ that minimizes the expected loss $\mathcal{L}\left(F\right):$, which is defined as ${\mathbb{E}}L(\mathcal{y},F(x))$. In this context, $L(\cdot , \cdot )$ represents a smooth loss function, and $(\text{x}, \mathcal{y})$ represents a $test example$ randomly selected from distribution $P$, entirely separate from the training dataset $\mathcal{D}$. CatBoost is a gradient boosting process that constructs a sequence of approximations ${F}^{t}: {\mathbb{R}}_{m}\to {\mathbb{R}}$ iteratively, starting from $t = 0$ and continuing in a greedy manner. To clarify, ${F}^{t}$ is derived from the preceding approximation ${F}^{t-1}$ in an additive manner: ${F}^{t}= {F}^{t-1} + \alpha {h}^{t}$, where $\alpha$ represents a step size, and function ${h}^{t}:{\mathbb{R}}_{m}\to {\mathbb{R}}$ (referred to as a base predictor) is selected from a set of functions $H$ to minimize the expected loss^34^.

3 $$\begin{array}{c}{h}^{t}=\underset{h\in H}{\text{arg }min}L\left({F}^{t-1}+h\right)=\underset{h\in H}{\text{arg }min}EL\left(\mathcal{y},{F}^{t-1}(\text{x})+h(\text{x})\right).\end{array}$$

Leveraging the aforementioned advantages, this study utilizes the machine learning algorithm CatBoost to discern the regression relationship of the CE across different cities in the YRD. Additionally, the latitude and longitude coordinates of each city are inputted into CatBoost as covariates in this study, aiding CatBoost in integrating spatial information for enhanced fitting.

Additionally, CatBoost enhances the interpretability of the model by calculating the importance of each feature based on the gain at each split node. During the training process, the algorithm records the gain (reduction in the loss function) for every feature each time it is used across all trees, accumulating these gains. Ultimately, the feature importance is normalized, representing the proportion of the total gain contributed by each feature to the overall gain. Specifically, gain can be defined as the difference in the loss function before and after a split using a particular feature. If the loss function before the split is $L$, and the loss functions after the split are ${L}_{\text{left}}$ and ${L}_{\text{right}}$, the gain can be expressed as:

4 $$\begin{array}{c}gain=L-\left( {L}_{\text{left}}+{L}_{\text{right}}\right).\end{array}$$

### Methods for algorithmic stacking—CatBoost-GWR (CGWR)

**Figure 4:**
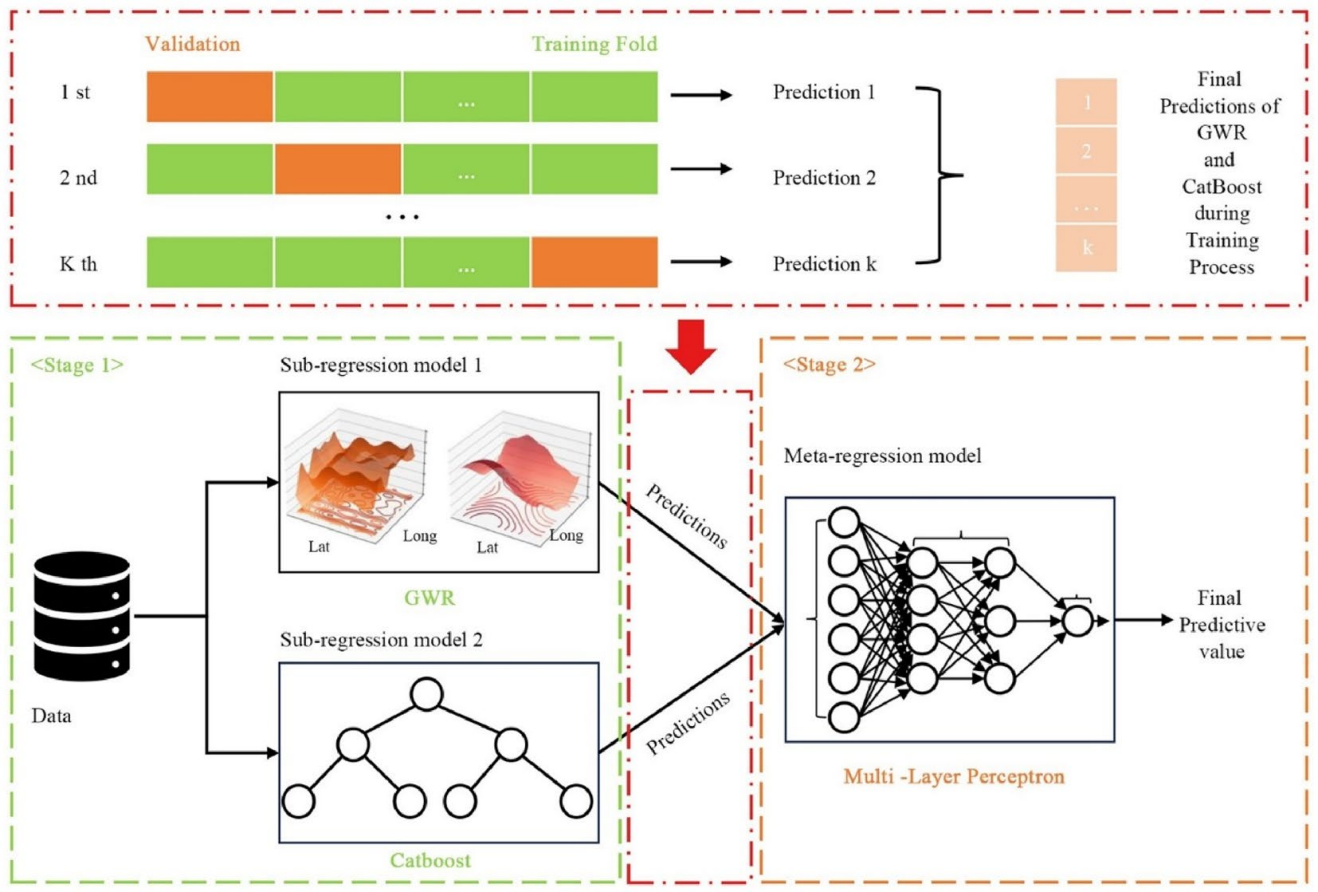
Stacked frame diagram of the improved spatial regression algorithm.

In this study, we construct a stacked model named CGWR by integrating the CatBoost algorithm with GWR. This enhanced spatial regression algorithm capitalizes on the robust interpretability of GWR for processing spatial data and the strengths of CatBoost in managing non-linearity, covariance, and small data samples. The objective is to enhance the data processing accuracy, predictability, and scalability of spatial analysis models, while ensuring their interpretability. The stacking framework introduced in this study is depicted in Fig. 4. The stacking ensemble method trains by leveraging the collective predictive outputs from multiple classification or regression models, which are then fed into a meta-classifier or meta-regressor^40^. For constructing a forecasting model, the initial or sub-level regression models are trained on the entire dataset. A meta-regression model is subsequently trained using these outputs as input variables. Stacked ensemble models typically surpass the performance of any individual model within the ensemble, effectively capitalizing on a diverse array of strengths^41^.

**Figure 5:**
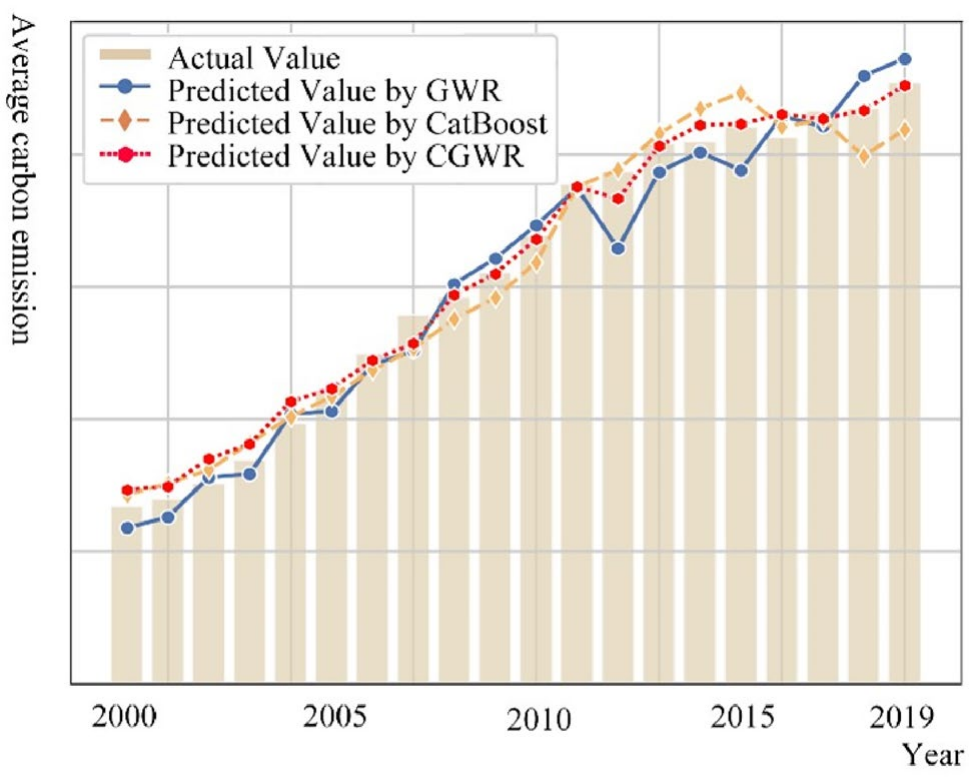
Comparison of average prediction results based on various spatial analysis algorithms with actual results (2000–2019).

**Figure 6:**
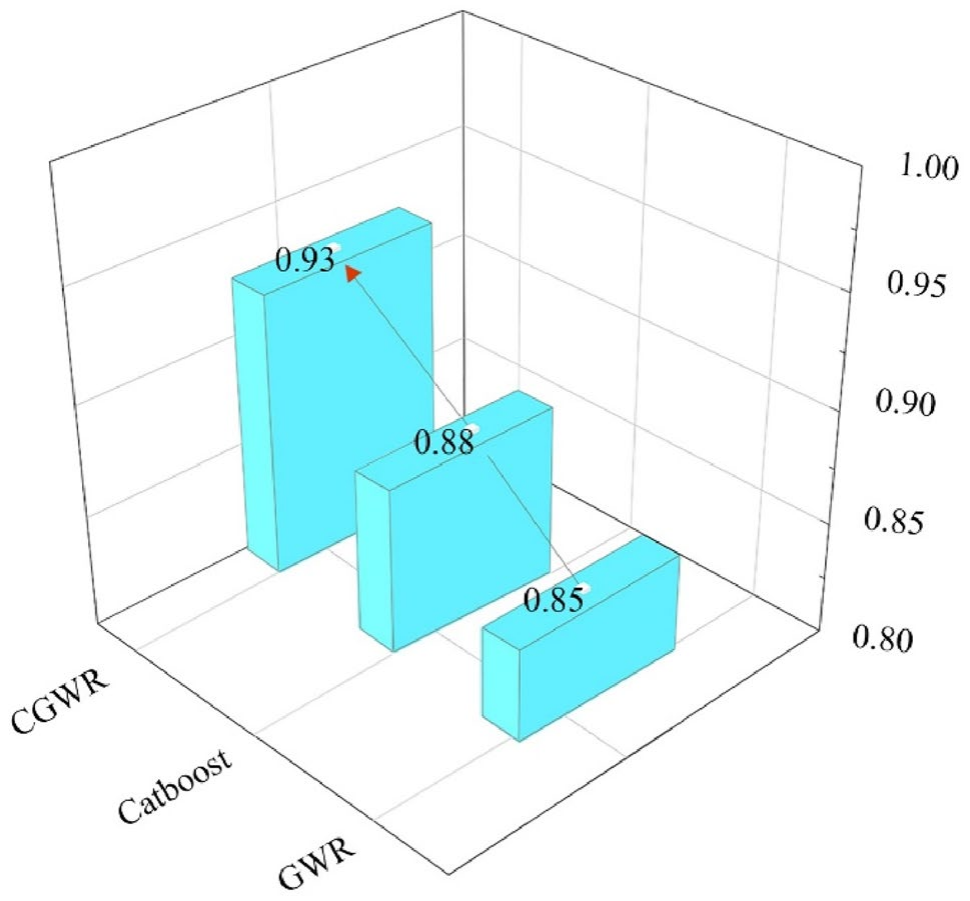
${R}^{2}$ comparison of spatial analysis models based on CE data of the YRD (2000–2019).

This study employs a cross-validation method to assess the model's efficacy. Specifically, GWR and CatBoost are deployed as sub-regression models, utilizing a fivefold cross-validation during the initial training phase to generate predictions. Throughout this process, the study consistently trains on four of the five folds for the GWR, CatBoost, and the integrative CGWR model. The models then predict outcomes for the remaining fold, a process repeated until all samples in the dataset are forecasted. Following this, a multi-layer perceptron is applied as a meta-regression model to delineate the functional relationship between the outputs of the sub-regression models and the final predictions. For new samples, predictions are initially generated using the sub-regression models trained on the complete dataset. These predictions are subsequently input into the meta-regression model to produce the final results. After testing its robustness and flexibility under various conditions, this paper conducts an empirical analysis of the spatiotemporal data on CE in the YRD region. Figure 5 displays a comparison of predictions made by various methods against actual data from 2000 to 2019, while Fig. 6 shows the fit of different algorithms over the same period. Notably, the GWR predictions considerably deviate from actual values in several instances. Although CatBoost, augmented with spatial information, shows reduced discrepancies compared to GWR, it remains less accurate than CGWR. Therefore, this section concludes that CGWR, an enhanced spatial analysis model that synergizes AI with traditional geospatial technologies, excels in processing capabilities. This approach effectively addresses the challenge posed in this article: accurately identifying critical elements and forecasting future scenarios while thoroughly considering the spatial interpretability and fit of CE data.

In this study, once the spatial regression relationship is established using the CGWR model, future values of the dependent variable can be forecasted by inputting the anticipated values of the independent variables. These independent variables are predicted utilizing the AutoRegressive Integrated Moving Average model, which is widely recognized as one of the most convinent and foremost autoregressive algorithms in machine learning. ARIMA is particularly effective for data that exhibit characteristics of time series, correlation, and non-linearity^42,43^.

## Results

### Spatio-temporal analysis of the CE in the YRD

**Figure 7:**
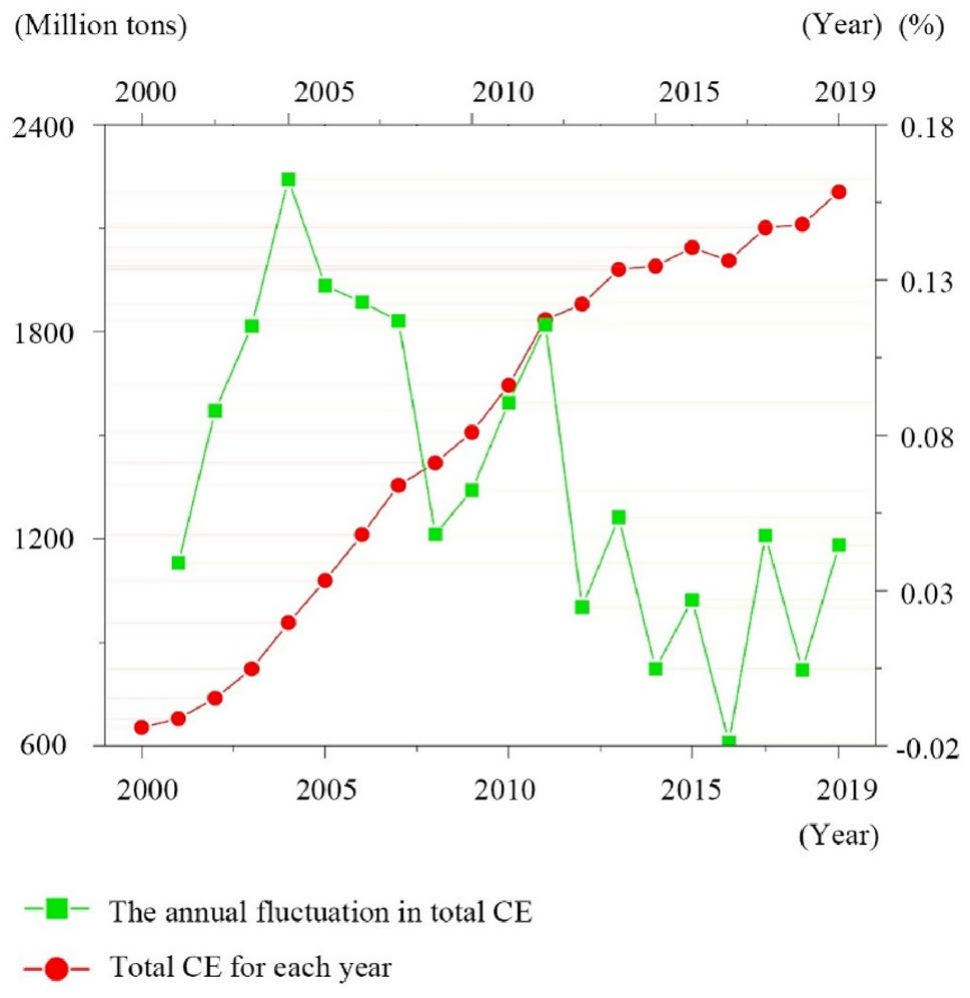
Year-by-year analysis for total CE of the YRD (2000–2019).

Figure 7 presents the annual total CE and their growth rates for the urban agglomeration in the YRD from 2000 to 2019. Since labeling each point with its specific value could clutter the graph, we use thin lines to help identify the exact values corresponding to each point. During the specified period, total CE in the YRD region escalated dramatically, rising from 653.38 million tons in 2000 to 2205.71 million tons in 2019. This surge accounted for nearly one-fifth of China's total CE during the same timeframe. Despite this significant increase, the growth rate of emissions has shifted from rapid to moderate, suggesting the initial effectiveness of CE management and control strategies in the region.

**Figure 8:**
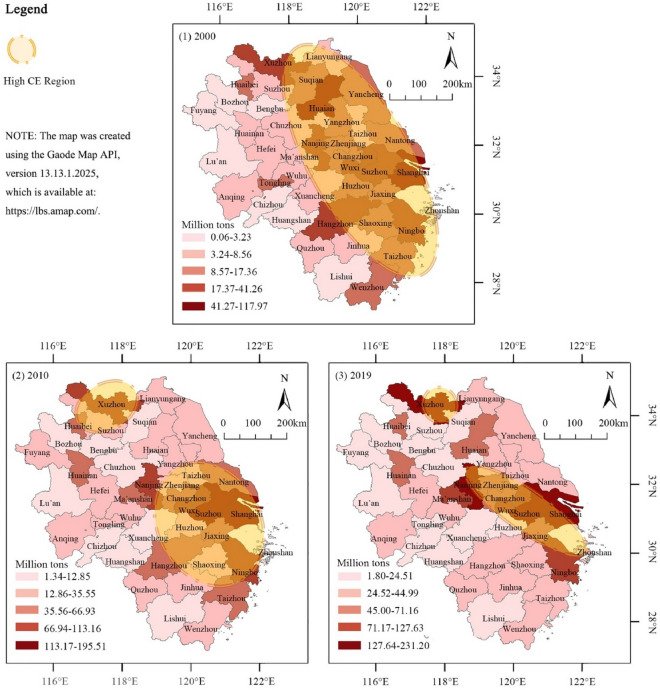
Spatial distribution analysis of the CE in the YRD (2000–2019).

Figure 8 employs the natural break method in ArcGIS 10.8 software to illustrate the spatial distribution of CE within the YRD urban agglomeration over these years. The analysis reveals a marked imbalance in the distribution of CE, with provincial capitals, southeastern cities, and northern cities exhibiting significantly higher emissions than other areas. This disparity is driven by several factors: Provincial capitals and southeastern cities, serving as economic and political centers, attract significant populations and resources, fostering rapid development in the industrial and service sectors and, consequently, higher energy consumption and CE. Northern cities, characterized by a predominance of heavy and energy-intensive industries, also report elevated CE levels. Additionally, long-standing policy biases and regional development strategies have further intensified these imbalances. It is notable that areas with high CE are increasingly concentrated in a few central-eastern cities within the YRD, marking a strategic shift in CE governance focus. By concentrating efforts on these key cities, enhancing their energy efficiency, promoting green energy initiatives, and optimizing industrial structures, effective control over the region's CE growth rate can be achieved. Moreover, this targeted approach is likely to enhance the regulatory influence and capacity of these critical cities over the entire region’s CE trajectory.

### Determining key factors affecting the CE from 2000 to 2019 based on CGWR

In the "Algorithm Stacking" section, we introduce the design principles of CGWR (Table 2) and its high-fitting validation results over the past 20 years (Figs. 5 and 6). In this study, while maintaining established parameters, the factor weights (feature importance) of each factor are computed through CGWR algorithm. This allows for an exploration of the weight relationships between CE and their impact factors for the years 2000, 2010, and 2019, as well as the variations in the weights of different elements over the past 20 years. The analysis of the weights of various factors is presented in Table 3. The impact factors represented by the code correspond to those listed in Table 1. Analysis using the CGWR model reveals that foreign investment, GDP, population, industrial particulate emissions, and sewage treatment rates are the principal contributors to CE within the YRD urban agglomeration from 2000 to 2019, collectively accounting for 63.24% of the emissions. Among these, GDP and foreign investment are indicative of economic activity levels, which drive industrial expansion and escalate energy demands, thus leading to an increase in CE. This trend highlights the YRD region's critical role in China's economic expansion and the potential environmental impacts of such heightened economic activities. Population growth, particularly in rapidly urbanizing areas, has also led to increased energy demands, primarily driven by residential and transportation needs. Additionally, the effects of industrial particulate emissions and sewage treatment rates underscore the crucial role of environmental governance in mitigating CE. The Table 2 suggests that enhanced environmental policies and governance can substantially reduce CE. Over time, the data also shows an increasing influence of energy consumption and human capital on CE, indicating that energy conservation and improvements in human capital quality are effective strategies for future CE reduction. This necessitates policymakers to align economic development goals with a stronger emphasis on energy efficiency and sustainable development, aiming to foster a symbiotic relationship between environmental stewardship and economic growth.

**Table 3:** Weights of factors on the CE in the YRD (2000–2019).

Code	2000 (%)	2010 (%)	2019 (%)	2000–2019 (%)
X1	4.34	8.84	4.30	8.51
X2	4.07	4.09	3.52	5.38
X3	7.87	9.78	22.58	9.91
X4	7.18	3.80	3.08	3.19
X5	4.49	6.28	4.05	1.70
X6	7.54	5.07	5.62	14.47
X7	4.10	12.43	7.75	4.31
X8	5.51	9.20	2.73	3.38
X9	6.08	5.56	2.78	15.92
X10	10.30	7.73	14.88	2.09
X11	6.18	6.72	11.20	3.58
X12	5.35	4.33	6.79	13.37
X13	3.04	4.97	5.32	3.66
X14	3.73	8.54	5.15	9.57
X15	20.22	2.67	0.23	0.96

### Projections for the CE based on CGWR (2025–2030)

**Figure 9:**
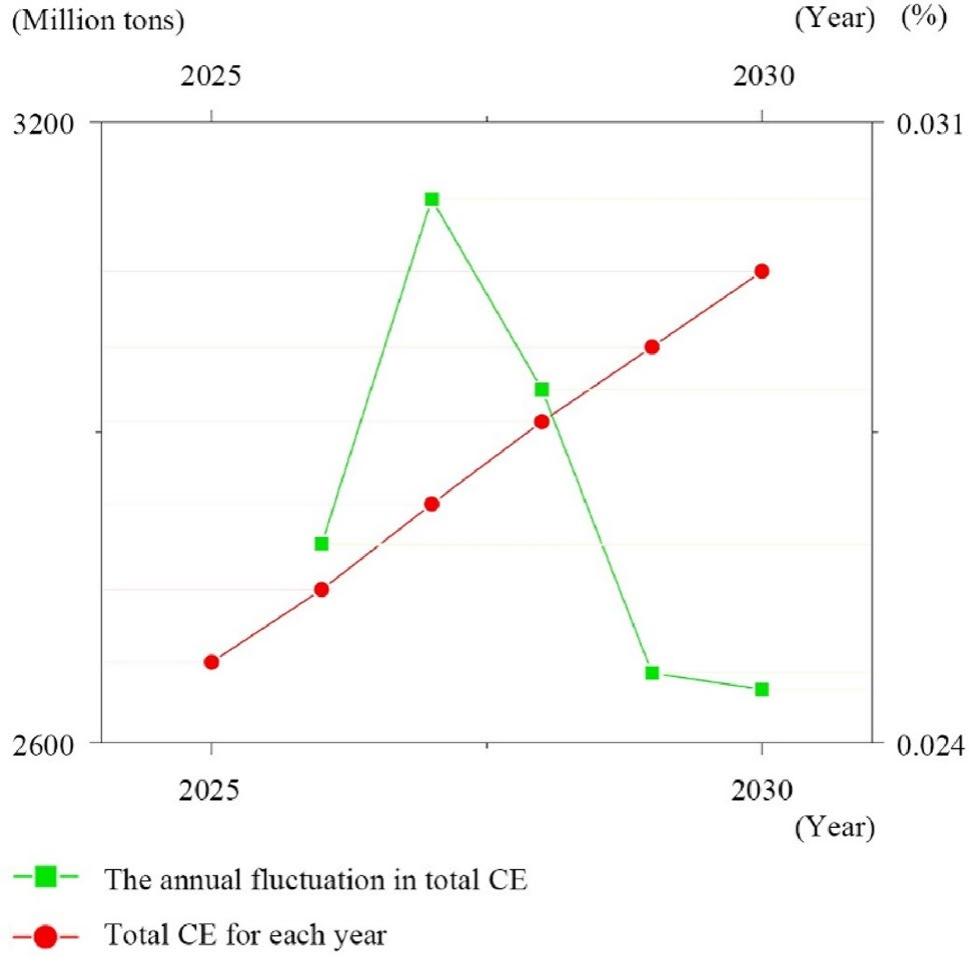
Future projections for the CE based on CGWR (2025–2030).

This study employs the CGWR model to project the trajectory of total CE in the YRD region from 2025 to 2030. The starting point of 2025 is chosen based on govement document predictions that Shanghai, as the economic heart of the YRD, will reach its peak CE in accordance with current carbon reduction policies and trends. The ending point of 2030 is in alignment with China's broader policy goals of achieving a carbon peak. Given the proactive nature of China’s carbon reduction efforts, this paper limits the projection period to maintain the accuracy of our predictions. The data suggests a continued, albeit significantly slowed, increase in the total CE of the YRD, estimated at 2677.89 million tons in 2025 and reaching 3055.66 million tons by 2030. The growth rate of CE is expected to be marginal, steadily approaching zero in 2030, as depicted in Fig. 9. This trend highlights the effectiveness of the carbon governance measures implemented in the region, suggesting the potential for the YRD to reach its CE peak before 2030.

As depicted in the figure, spatial variations among key cities from 2025 to 2030 are minimal, suggesting a stabilization in the spatial structure. This is a change from the period between 2000 and 2019, during which two distinct high-CE belts emerged: one centered around provincial capitals and another around heavy industrial cities in the northeast. The formation and persistence of these belts can be attributed to several factors: firstly, provincial capitals, as economic and administrative centers, naturally attract significant economic activities and populations, leading to higher energy consumption and CE. Secondly, cities in the northeast with a heavy industrial base, including industries like steel and chemical manufacturing, show higher CE levels due to the nature of their energy-intensive industries. Lastly, a spatial Matthew effect is observed in cities with high CE, where economically developed and higher-emitting cities tend to attract additional investments and resources, further increasing their carbon footprints. The forecasts and analyses in this study highlight two critical directions for future spatial management of CE in the YRD: concentrating efforts on the identified high-CE belts and enhancing regional coordination and planning across provincial administrative boundaries. This approach necessitates policymakers not only to formulate and implement carbon reduction strategies at the provincial level but also to strengthen inter-regional cooperation to collectively tackle CE challenges, aiming to achieve the YRD’s carbon peak goals by 2030.

## Conclusion

In the context of global warming, this study employs advanced spatial analysis techniques to provide a more precise delineation of the spatial distribution of CE and its key driving factors in the YRD region for the periods 2000–2019 and 2025–2030. The key findings are as follows:

1. Trend analysis: The YRD urban agglomeration exhibited a general trend of increasing CE, with the rate of increase gradually approaching zero. Predictive analysis suggests a high likelihood of the YRD achieving its CE peak before 2030, facilitated by strategic policy adjustments. Spatially, the region has developed two significant high CE belts concentrated around provincial capitals and industrial cities in the northeast.
2. Fit quality of spatial analysis: In terms of fit quality of spatial analysis techniques, the Combined CGWR model demonstrated superior performance with a fit of 0.93, compared to CatBoost at 0.88 and GWR at 0.85. Empirical results indicate that the integration of AI with traditional geospatial technologies in urban spatial analysis not only improves the balance between data processing quality and spatial interpretability but also enhances the overall fit. Moreover, CGWR exhibits increased practicality, predictability, scalability, and comparability.
3. Key factors influencing CE: Identifying the key factors influencing CE in the YRD region is crucial for the formulation of effective policies. The current analysis indicates that a balanced approach among economic development, human capital, governance capabilities, and energy consumption is essential for expediting the YRD's achievement of its carbon peak. Among these key factors, the economic foundation has been identified as the most significant influence on the total CE in the YRD from 2000 to 2019, with GDP and actual foreign investment playing particularly prominent roles, accounting for 14.47% and 15.92% of the influence, respectively. In the context of regional CE control, these factors warrant special attention.

## Discussion

**Figure 10:**
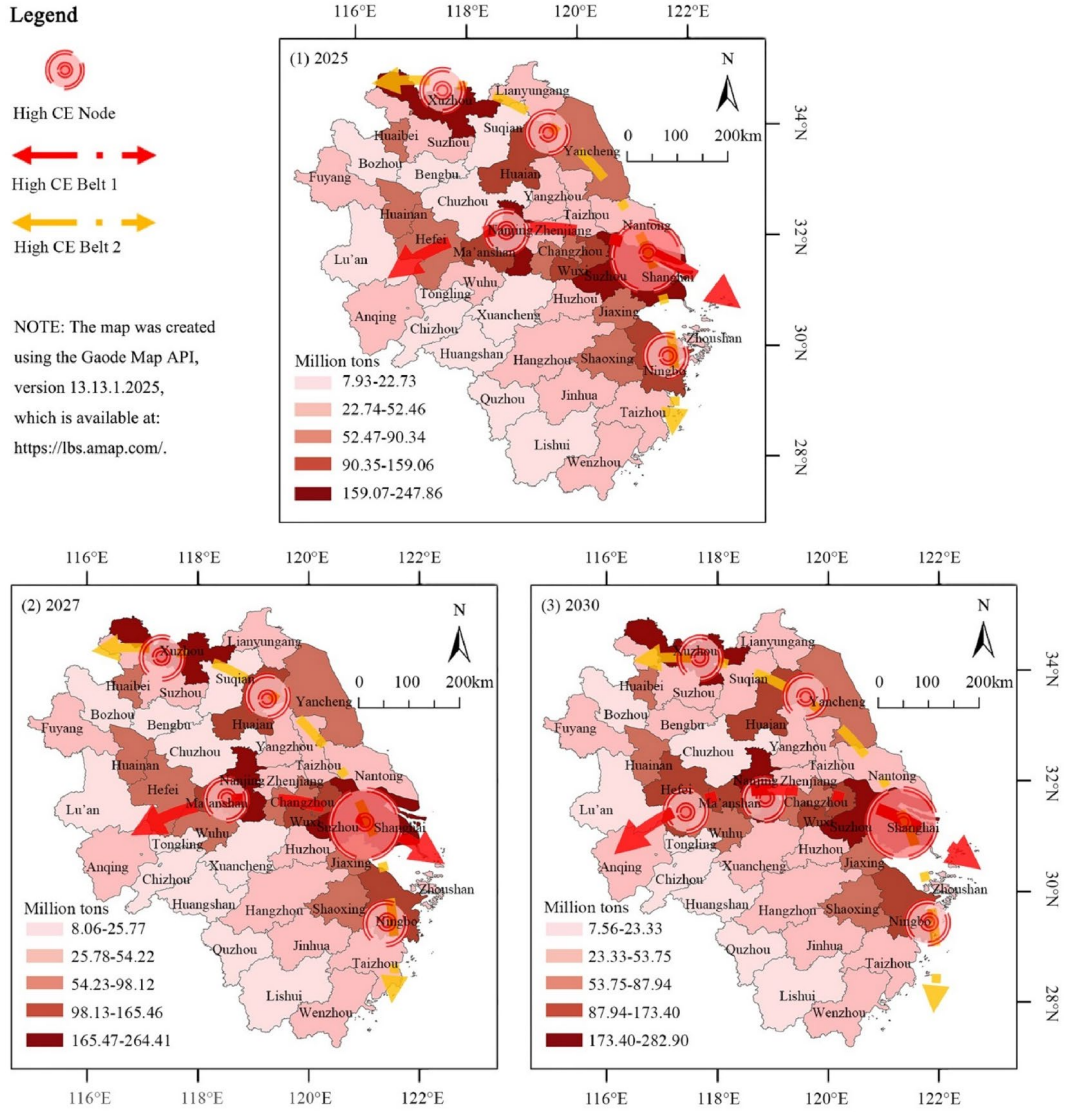
Predicted spatial distribution of CE in the YRD by CGWR (2025–2030).

This paper utilizes AI to clarify and predict urban CE patterns directly related to climate change. By employing advanced spatial analysis techniques, this study provides a detailed characterization of the spatial distribution of CE and its key determinants in the YRD region for the periods 2000–2019 and 2025–2030. The following points are highlighted for further discussion:

1. Integration of AI and traditional geospatial technologies: Traditional geographical analysis methods, such as GWR and Geodetector, are robust in spatial interpretation but rely on OLS to fit linear relationships, which are less effective with non-linearities, covariance, and small sample sizes typical of urban CE data. AI technologies, capable of superior fitting with high-dimensional data, are often limited by weaker spatial interpretability. This study innovatively combines the strengths of AI with traditional geospatial technologies to develop the CGWR algorithm (Table 2). Experiments have shown that CGWR not only improves data processing capabilities but also maintains spatial interpretability and ensures high accuracy (Figs. 5 and 6). CGWR provides a valuable tool for government decision-makers to assess spatial distribution across cities and devise targeted regional sustainable development strategies. This study also opens new avenues for incorporating multifaceted considerations and invites collaboration with other researchers to further refine the functionalities and accuracies of spatial analysis models, including temporal dimensions and spatial identification techniques.
2. Refining for future prediction: The primary goal of this study is to enhance the spatial analysis of CE in the YRD region, leading to macro-level recommendations that are closely related to the regional status quo (Fig. 10 and Table 3). And the CGWR method can also facilitate customized analyses and predictive solutions for different provinces and cities, enabling more specific and detailed forecasts. Future researchers are encouraged to assess variations in CE levels across different cities under various planning strategies, using the methodologies and perspectives from our study to formulate optimized urban sustainable development strategies. Considering the reliability and comprehensiveness of data collection, this study selected data from 2000 to 2019. With standardized regional CE metrics, ongoing disclosure of official data, and advancements in CE calculation methods, conducting long-term tracking studies on the spatial distribution of CE is highly feasible and anticipated.
3. Temporal and spatial changes in CE: Investigation of temporal and spatial changes in CE in the YRD region projects that total emissions are expected to reach 3055.66 million tons by 2030, with the growth rate nearing zero (Figs. 9 and 10). This projection supports the expectation that the YRD will achieve its CE peak by 2030, underscoring the region's significant role and the long-term research value of the YRD urban agglomeration in ongoing and future carbon reduction efforts. Notably, the spatial distribution of CE in the YRD is expected to remain imbalanced by 2030, with clear trends in the development of the spatial structure of CE, particularly with the emergence of high CE belts. This indicates that further research is necessary on the structural formation of high CE areas, the spatial correlations of CE between cities, major influencing factors, and their prospective development trends..

## Recommendations

To advance carbon reduction and sustainable development within the cities of the YRD, the following strategies are proposed, based on the findings of this study and the distinct characteristics of the YRD urban agglomeration:

1. Explore new economic growth paradigms for the YRD: The economic foundation significantly impacts CE in the YRD. In response to emerging challenges such as the climate crisis, slowing economic growth, and diminishing demographic dividends, the YRD must develop new economic growth strategies. Opportunities include transforming the region into a new hub for international economic, social, and cultural exchanges, upgrading industrial chains, emphasizing innovation, and engaging with the "Belt and Road" initiative to open new avenues for economic development.
2. Strengthen spatial linkages between cities in the YRD: This study highlights substantial spatial autocorrelation and clustering of CE within the YRD, with some cities interacting across different provincial administrative boundaries. This observation underscores the need for a unified regional coordination plan to pursue shared low-carbon development goals. Efforts should focus on enhancing regional cooperation in low-carbon initiatives, concentrating control in high CE areas, and seeking a balance between urban carbon control and regional carbon management. The governments of the YRD should play a central role in formulating and implementing consistent regional policies and leverage the influence of dominant cities within each province to catalyze transformation in less developed areas through synergistic and complementary strategies.
3. Raise public awareness of green consumption in the YRD: The population plays a crucial role in urban development and significantly influences CE. Advocating for resource conservation, recycling, and promoting a green, low-carbon lifestyle are vital. The YRD should accelerate the development of low-carbon communities and launch comprehensive campaigns to promote low-carbon lifestyles. This involves translating broad low-carbon objectives into specific emission reduction strategies for individual residents, thereby fostering community-wide engagement in sustainability efforts.

## Data Availability

The data presented in this study are available on request from the corresponding author. The data are not publicly available due to an ongoing study.
